# Improved access to the bone marrow space by multiple perforations of the alveolar bundle bone after tooth extraction—A case report

**DOI:** 10.1002/cre2.474

**Published:** 2021-07-23

**Authors:** Christian Ulm, Georg D. Strbac, Andreas Stavropoulos, Azadeh Esfandeyari, Toni Dobsak, Kristina Bertl

**Affiliations:** ^1^ Division of Oral Surgery, University Clinic of Dentistry Medical University of Vienna Vienna Austria; ^2^ Department of Periodontology, Faculty of Odontology University of Malmö Malmö Sweden; ^3^ Division of Regenerative Dentistry and Periodontology, University Clinics of Dental Medicine (CUMD) University of Geneva Geneva Switzerland; ^4^ Division of Conservative Dentistry and Periodontology, University Clinic of Dentistry Medical University of Vienna Vienna Austria; ^5^ Core Facility Hard Tissue and Biomaterial Research, Karl Donath Laboratory, School of Dentistry Medical University of Vienna Vienna Austria

**Keywords:** alveolar bundle bone, blood supply, cortical bone, extraction socket healing, perforation, ridge preservation

## Abstract

**Objectives:**

The dental alveolus is lined by a thin cortical layer (“bundle bone”, “alveolar bone proper”, “cribriform plate”, “lamina dura”), that can impede access to the bone marrow and its vasculature. During unassisted socket healing, the alveolar bundle bone is gradually resorbed allowing tissue resources from the bone marrow to enter into the socket space. An optimized wound healing process, either during unassisted socket healing or during ridge preservation procedures, with autogenous bone and/or any bone/collagen substitute material, depends at least partly on an adequate vascularization of the socket space. This ensures sufficient recruitment of osteoblast and osteoclast precursor cells and facilitates fast bone regeneration and/or uneventful integration of the augmentation material.

**Methods:**

The present technical note describes an easy treatment step after tooth extraction aiming to improve socket healing with or without any ridge preservation procedure, by facilitating an increased blood inflow into the dental alveolus. Specifically, after tooth extraction the alveolar bundle bone is perforated several times – mainly in a palatally/lingually – by a small round bur (diameter < 1 mm) extending into the trabecular bone.

**Results and conclusions:**

By means of this relatively simple treatment step, an increased blood inflow into the alveolus is achieved after tooth extraction, which might enhance socket healing and corticalization of the entrance, and in turn result in a lower complication rate (e.g., dry socket), in an enhanced graft incorporation, and/or in a reduced loss of alveolar ridge volume.

## 1 INTRODUCTION

**Figure 1:**
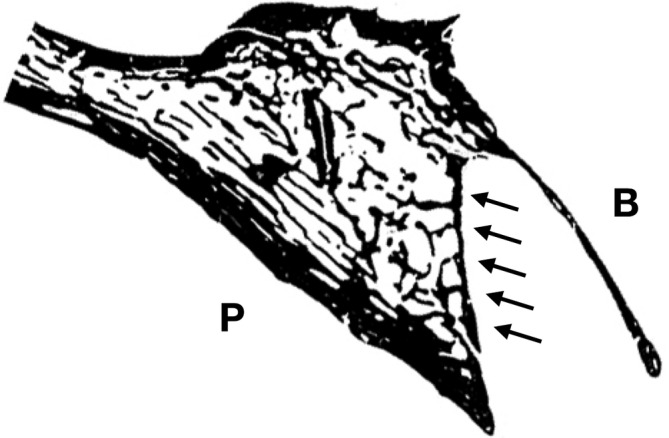
Human histologic section of the upper lateral incisor (P, palatal; B, buccal; modified Von Kossa staining, anatomic collection of the authors). On the palatal side, the thin cortical bone layer of the alveolar bone proper separates the empty dental alveolus from the bone marrow of the retroalveolar trabecular bone (black arrows). On the buccal side, the alveolar bone proper is fused with the external cortical plate of the alveolar process

Physiologically, the wall of the dental alveolus mostly consists of a thin cortical bone layer (also called “bundle bone,” “alveolar bone proper,” “lamina dura” or “cribriform plate”), with a thickness ranging from 0.22 to 0.54 mm (Hubar, 1993), depending on the region (Figure 1). At the incisors and canines, the bundle bone of the labial surface often fuses with the external cortical plate of the alveolar process, whereas on the palatal/lingual side regularly a varying volume of trabecular bone is present between the bundle bone and the inner cortical plate of the alveolar process (“retroalveolar bone”; Sicher & Du Brul, 1975).

**Figure 2:**
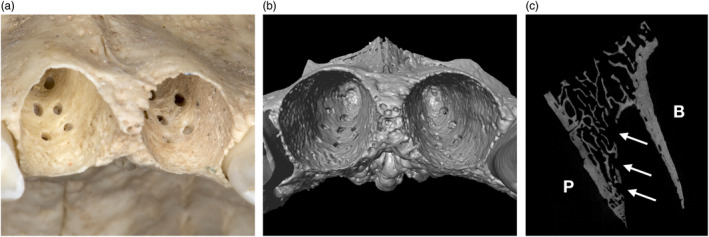
(a) Human maxillary cadaveric specimen showing two empty central incisal alveoli after making multiple perforations of the palatal bundle bone in relatively close proximity to each other (i.e., 2–3 mm distance in‐between); (b) occlusal view of a micro computed‐tomography scan of the same specimen displaying about 10 small perforations in each socket; (c) cross‐sectional slice of the same scan illustrating three palatal perforations of the alveolar bone proper extending into the trabecular bone (white arrows). B, buccal; P, palatal

After tooth extraction the dental alveolus regularly suffers loss in height and width (Sculean et al., 2019); a systematic review described a horizontal bone loss of 29%–63% (corresponding to 2.5–4.5 mm) and a vertical bone loss of 11%–22% (corresponding to 0.8–1.5 mm) for unassisted socket healing within the first 6 months (Tan et al., 2012). Thereafter, these changes in ridge dimension appear to slow down but continue throughout life (Carlsson & Persson, 1967). In order to reduce this volume reduction of the alveolar ridge, numerous ridge preservation techniques have been described; a significant overall reduction of vertical bone loss has been described with these approaches, but relatively limited reduction—and with large variation—of horizontal bone loss compared to unassisted socket healing. Thus, no specific technique can be recommended as superior based on the available evidence (MacBeth et al., 2017; Mardas et al., 2015). In this context, during guided bone regeneration (GBR) and block augmentation procedures, several small perforations of the cortical bone at the recipient site (i.e., decortication) are recommended to open the bone marrow space, increase the blood inflow, and allow migration of osteogenic tissue resources into the defect area (Cha et al., 2012; Lee et al., 2014); such perforations have been also suggested for the sinus floor during sinus‐lift procedures (Ulm et al., 2017). More specifically, in the context of GBR procedures the process of new bone formation follows a specific sequence, that is, the initially formed blood clot is resorbed by neutrophils and macrophages and replaced by granulation tissue within a few days. In order to achieve in the next steps unmineralized bone (i.e., osteoid), woven bone, and finally lamellar bone, numerous blood vessels transporting progenitor cells and nutrients are required, that is, the creation of new blood vessels regularly originates from pre‐existing blood vessels and precedes bone formation. Decortication of the recipient site offers access to the more vascular trabecular bone, promotes bleeding, blood clot formation, release of growth factors and cytokines, and finally results in the recruitment of pro‐angiogenic and pro‐osteogenic cells (Greenstein et al., 2009). The cortical character of the bundle bone of an empty dental alveolus may be seen in a similar manner as the cortical layer at the recipient site during GBR; hence, as in the above‐outlined reasoning for decortication during GBR procedures, perforations of the palatal/lingual wall after tooth extraction (Figure 2) might result in an improved unassisted socket healing and corticalization of the entrance (Bertl et al., 2018; Trombelli et al., 2008). Furthermore, it may result in enhanced bone formation and/or incorporation of any grafting material, in turn resulting in reduced reduction of the alveolar ridge volume (i.e., improved ridge preservation; Mardas et al., 2015). With this case report, the possibility to make perforations in the bundle bone of an extraction socket, aiming to improve socket healing by facilitating an increased blood inflow, is suggested.

## 2 CASE REPORT AND DESCRIPTION OF THE TECHNIQUE

**Figure 3:**
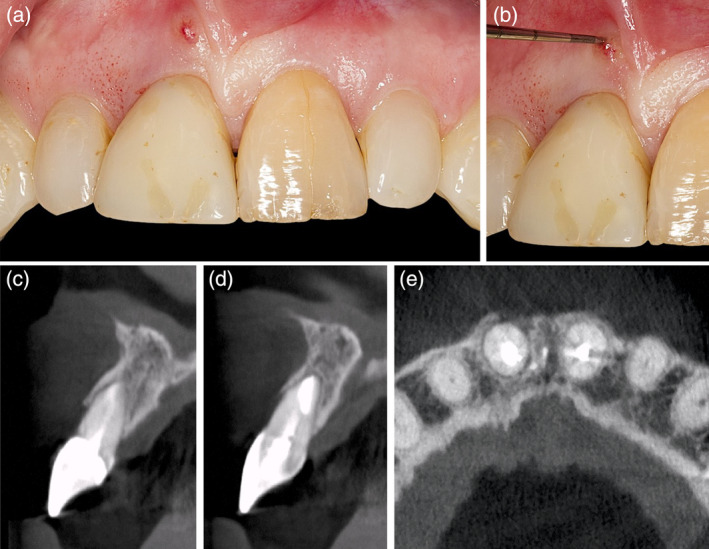
(a) and (b) clinical case with an endodontically treated but failing tooth in position #11 with a buccal fistula; (c)–(e) the computed tomography confirmed the buccal bone resorption in the region of the fistula

**Figure 4:**
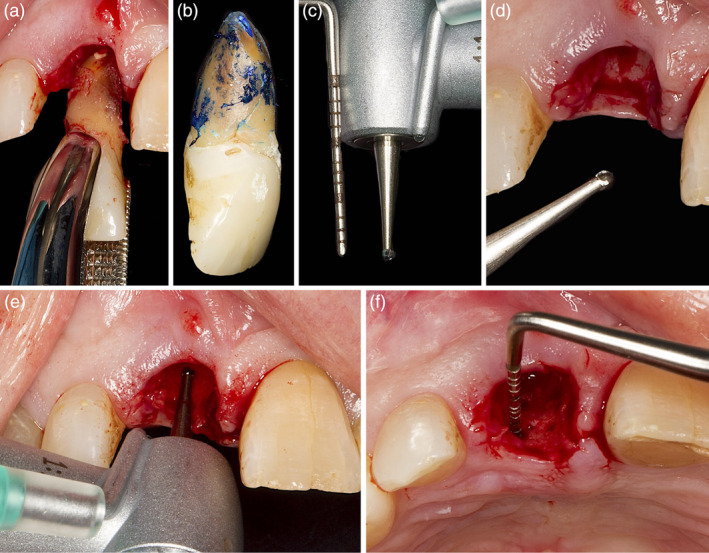
(a) After careful tooth extraction the vertical fracture line along the root became evident (b); (c)–(f) several perforations in the palatal aspect of the bundle bone were made with a small round bur with a diameter <1 mm

**Figure 5:**
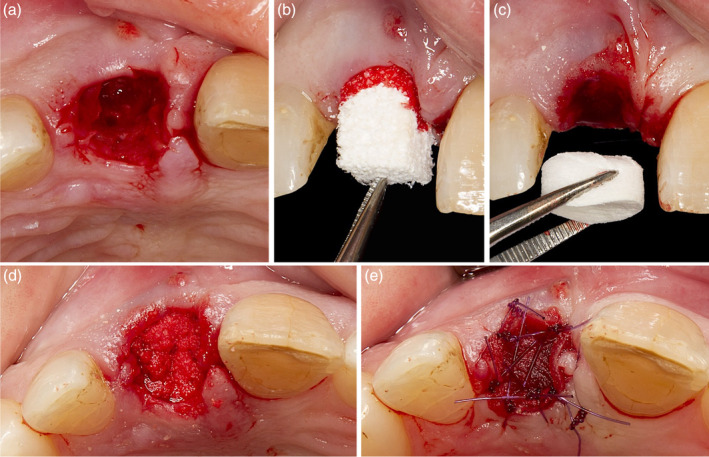
(a) Depending on the size of the extraction socket, about 6–10 small perforations are made prior to ridge preservation procedure; (b) a combination product of bovine‐derived xenograft and porcine‐derived collagen (Bio‐Oss® Collagen, Geistlich Pharma AG, Switzerland) was used for grafting of the extraction site and the socket entrance was sealed with a porcine collagen matrix (Mucograft® Seal, Geistlich Pharma AG, Switzerland; c–e)

**Figure 6:**
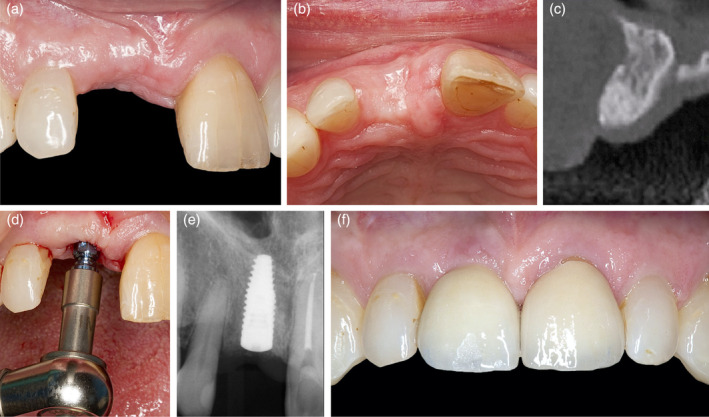
(a) and (b) Clinical and (c) radiographical situation after a healing period of 9 months prior to implant installation (d) and (e) and prosthetic restoration (f)

The patient presented with a failing tooth in position #11 (Figure 3). After careful tooth extraction the additional treatment step was performed, that is, perforations in the palatal aspect of the bundle bone with a small round bur with a diameter <1 mm (Figure 4). Thereafter, the socket was grafted with a combination product of bovine‐derived xenograft and porcine‐derived collagen (Bio‐Oss® Collagen, Geistlich Pharma AG, Switzerland) and the socket entrance was sealed with a porcine collagen matrix (Mucograft® Seal, Geistlich Pharma AG, Switzerland; Figure 5). After a healing period of 9 months a bone level type implant (BLT, SLActive®, 4.1 × 12 mm, Institut Straumann AG, Switzerland) was installed and restored with a single crown (Figure 6).

This additional treatment step can be performed after tooth extraction independent whether unassisted socket healing or any ridge preservation procedure is planned. Depending on the size of the extraction socket, about 6–10 small perforations of the palatal/lingual and in the absence of neighboring teeth of the mesial/distal aspect of the bundle bone are made in relatively close proximity to each other (i.e., 2–3 mm distance in‐between; Figure 2, 4, and 5(a)). The perforations should extend into the trabecular bone to provoke bleeding, but care should be taken not to drill too deep in order to maintain the integrity of the external plate of the alveolar process at the palatal/lingual aspect. The thickness of the palatal/lingual bundle bone can be either determined—if available—on pre‐operative (cone beam) computed tomography scans or estimated by assessing the thickness of the palatal soft tissue (e.g., with the needle during local anesthesia or by bone sounding with a periodontal probe). On the buccal side, especially in the anterior regions of the jaws, the bundle bone is most often fused with the external plate of the alveolar process (Sicher & Du Brul, 1975) and ≤1 mm thick (Tsigarida et al., 2020), and thus this aspect of the socket is mainly composed of cortical‐ and only minor trabecular bone, explaining the increased resorption observed from this aspect; thus, the buccal bundle bone should be spared, and no perforations should be made. In general, this technique could be performed in any region of the upper and lower jaw, taking the specific anatomic conditions of each region, as well as relevant anatomic variations, into account (López‐Jarana et al., 2021; Park et al., 2014). After all perforations are made any further treatment steps for ridge preservation may be continued (i.e., hard and/or soft tissue grafting, wound closure, etc.).

## 3 DISCUSSION

The above‐described additional simple treatment step after tooth extraction is based on the rationale that one major factor during bone healing and grafting material incorporation is sufficient vascularization, which provides the necessary cells and healing factors (Cha et al., 2012; Lee et al., 2017; Schmid et al., 1997); disturbance or lack of vascularization may result in delayed healing and/or compromised integration of the grafting material (Degidi et al., 2007). Preclinical and clinical trials have provided histological evidence of the relevance of sufficient capillary ingrowth for new bone formation and, consequently, of the positive effect of such perforations during GBR and block augmentation procedures; that is, faster incorporation and/or reduced resorption of bone grafts, and larger amounts of bone gain, after perforation of the cortical layer of the recipient site compared to cases without perforations has been observed (Greenstein et al., 2009). For example, in a rabbit GBR model, perforations of the recipient bed improved neo‐angiogenesis in bone grafts and increased neo‐osteogenesis, especially in the early healing phase (Lee et al., 2014), while in a recent human GBR study a positive effect of cortical bone perforations on angiogenesis and osteogenesis, in terms of histomorphometrically confirmed increased new bone formation within the regenerated alveolar process, was observed (Danesh‐Sani et al., 2017). Further, a randomized controlled clinical study showed that perforations of the bone wall in 2‐ and 3‐wall intrabony periodontal defects, during open flap debridement, resulted in significant clinical and radiographical bone gain compared to open flap debridement alone (Crea et al., 2014). In this context, pre‐clinical studies have shown that use of bone substitute materials, may in fact delay bone formation, especially under optimal conditions of space provision (Aroni et al., 2019; Stavropoulos et al., 2001; Stavropoulos et al., 2003). Since ridge preservation procedures include in many cases the application of bone substitutes, a simple additional procedure as the one described herein, could be beneficial in terms of enhancing bone regeneration and integration of the grafting material. Perforation of the cortical alveolar bundle bone, except from facilitating socket vascularization and access to the tissue resources of the bone marrow in the trabecular compartment of the alveolar ridge, may additionally enhance socket healing by enhancing local bone remodeling, a mechanism known as Frost's “regional acceleratory phenomenon” (Frost, 1983).

The proposed surgical technique with a small bur is simple to incorporate into the clinical procedure as the perforations of the bundle bone are made within only a few seconds. Up to now, several patients have been treated using this modified ridge preservation technique at the Division of Oral Surgery (University Clinic of Dentistry, Medical University of Vienna, Austria). No complications have been observed in terms of the access to make the perforations neither in terms of any conceivable post‐surgical events related to the modification (e.g., excessive hematoma, wound dehiscences, etc.); further, the procedure was well accepted by the patients.

Of course, the present report represents only a single case without any histological assessment and/or comparison to a control (non‐perforated) socket. Hence, the lack of standardized/controlled assessment of this technique—up to now—does not allow any conclusion on whether perforations of the alveolar bone proper actually result in any tangible clinical or histological benefit; for example, reduced rate of dry sockets, enhanced bone formation and corticalization, and/or graft incorporation resulting in reduced alveolar ridge resorption, and so on. Preclinical trials aiming for micro computed‐tomography and histological assessment of the outcome have been launched to assess the effect size of this simple modification during alveolar ridge preservation procedures.

## CONFLICT OF INTEREST

The authors declare that they have no conflict of interests.

## AUTHOR CONTRIBUTIONS

*Concept/design*: Christian Ulm, Georg D. Strbac, Andreas Stavropoulos and Kristina Bertl. *Data collection of the articl* *e*: Christian Ulm, Georg D. Strbac, Azadeh Esfandeyari, Toni Dobsak and Kristina Bertl. *Drafting article*: Christian Ulm, Andreas Stavropoulos and Kristina Bertl. *Approval of the article*: Christian Ulm, Strbac, Andreas Stavropoulos, Azadeh Esfandeyari and Kristina Bertl.

## ETHICS STATEMENT

Ethical approval was given by the ethics committee of the Medical University of Vienna (EK‐Nr. 1709/2020).

## Data Availability

Data available on request from the authors.
